# Editor's Note: 2013 Reviewers of the Year

**DOI:** 10.1289/ehp.122-A37

**Published:** 2014-02-01

*EHP* received more than 1,500 submissions during 2013, and nearly 500 of those papers were sent out for peer review. Each paper is evaluated by at least 2 reviewers for scientific quality and content. *EHP* is very grateful to the nearly 1,000 reviewers who were part of the peer-review process. A list of those reviewers is available on the journal’s website. *EHP* would also like to recognize our top Reviewers of the Year. These individuals completed at least 5 reviews during the year and received high ratings from the Associate Editors for the timeliness and quality of their reviews. The *EHP* 2013 Reviewers of the Year are Daniel Axelrad, John Balmes, Aaron Barchowsky, Adrian Barnett, David Bellinger, Joe Braun, Gerard Hoek, Matthew Longnecker, and C. Arden Pope III. *EHP* is grateful to these and all other reviewers who assisted us during 2013.

